# Supplementary figures and images for: Spatio-temporal distribution of Spiroplasma infections in the tsetse fly (Glossina fuscipes fuscipes) in northern Uganda
Source: PLoS Negl Trop Dis. 2019 Aug 1;13(8):e0007340. doi: 10.1371/journal.pntd.0007340 (PMC6692048; doi:10.1371/journal.pntd.0007340)

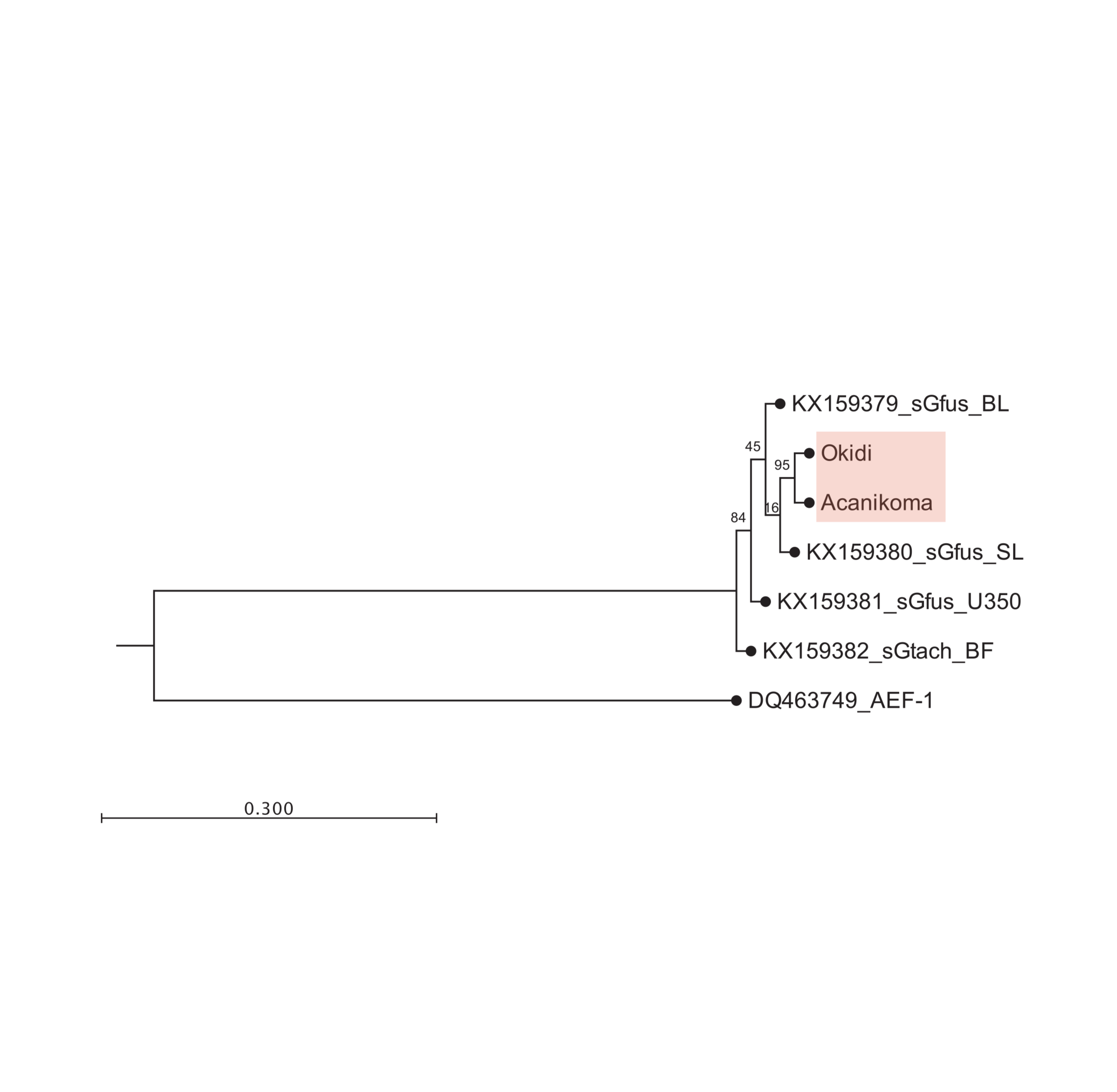

Supplement: S1 Fig — Spiroplasma strains from the Albert Nile watershed (OKS) and the Okole River watershed (ACA) are identical to each other and highly conserved in comparison to the Spiroplasma present in two individuals screened from a Gff laboratory line (KX159379_sGfus_BL, KX159380_sGfus_SL) and one Uganda field sample (KX159381_sGfus_U350). These sequences were recently published in [31]. The tree was calculated in CLC using Jukes-Cantor as distance measure with 1000 replicates, and rooted against the outgroup Spiroplasma culicicola. Scale is 0.3 substitutions per site. (TIF) [file pntd.0007340.s001.tif]

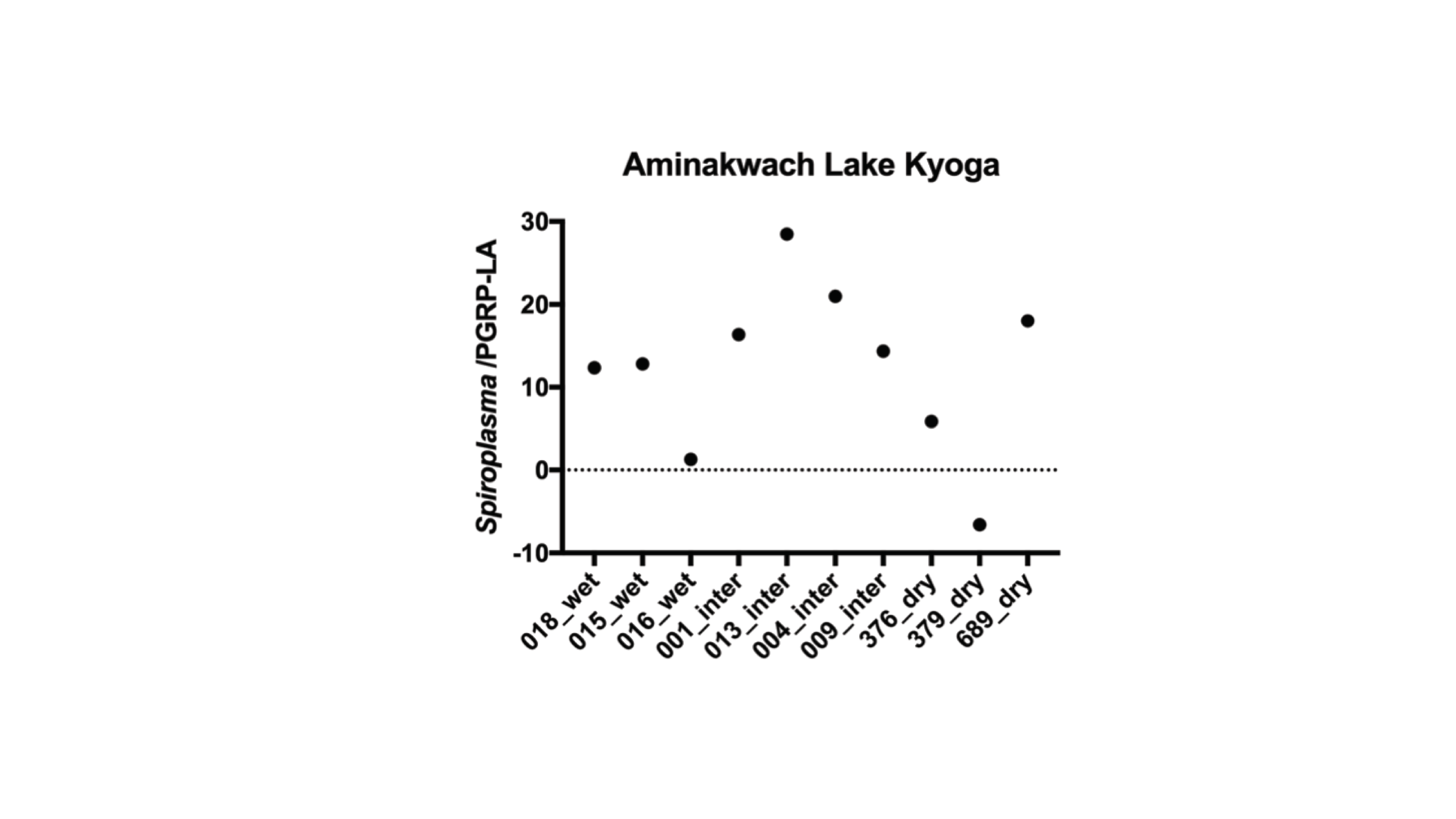

Supplement: S2 Fig — The graph shows the relative density of Spiroplasma tested via 16S rDNA in Gff from AMI population (Lake Kyoga) across wet, dry and intermediate season. Spiroplasma levels are higher in intermediate and wet season compared to the dry season. (TIF) [file pntd.0007340.s002.tif]

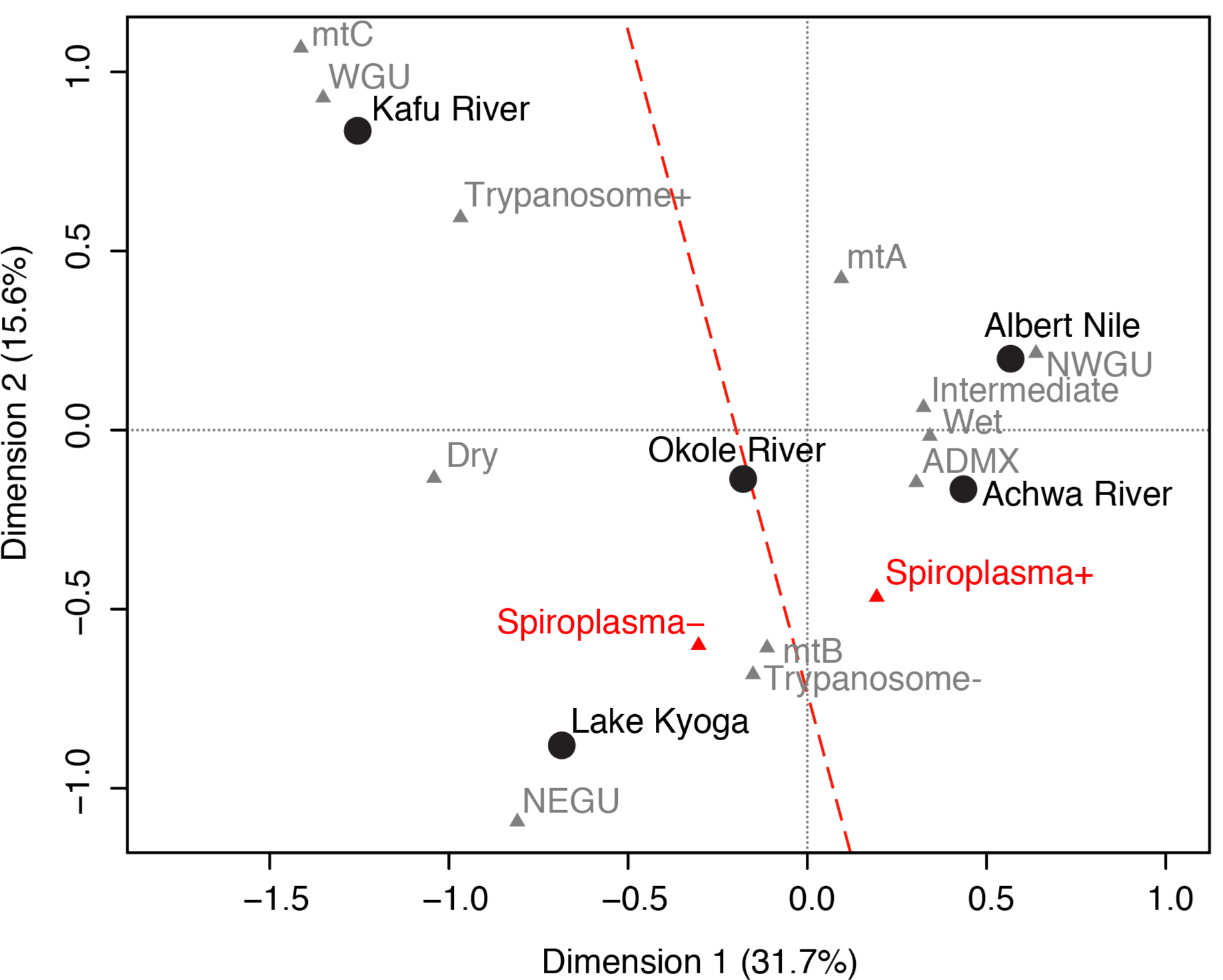

Supplement: S3 Fig — Dimensions 1 and 2 (accounting for 31.7% and 15.6% of the variation) are plotted. Populations are depicted by triangles. Association with trypanosomes and Spiroplasma is shown as open triangles (infected and uninfected). Further associations shown in the plot are the host mtDNA genetic background (mtA, mtB, mtC), the nuclear genetic background (NWGU, ADMX, NEGU, WGU), the season (Dry, Inter, Wet), and the watershed of origin (Albert Nile, Achwa River, Okole River, Lake Kyoga, Kafu River). (TIF) [file pntd.0007340.s003.tif]

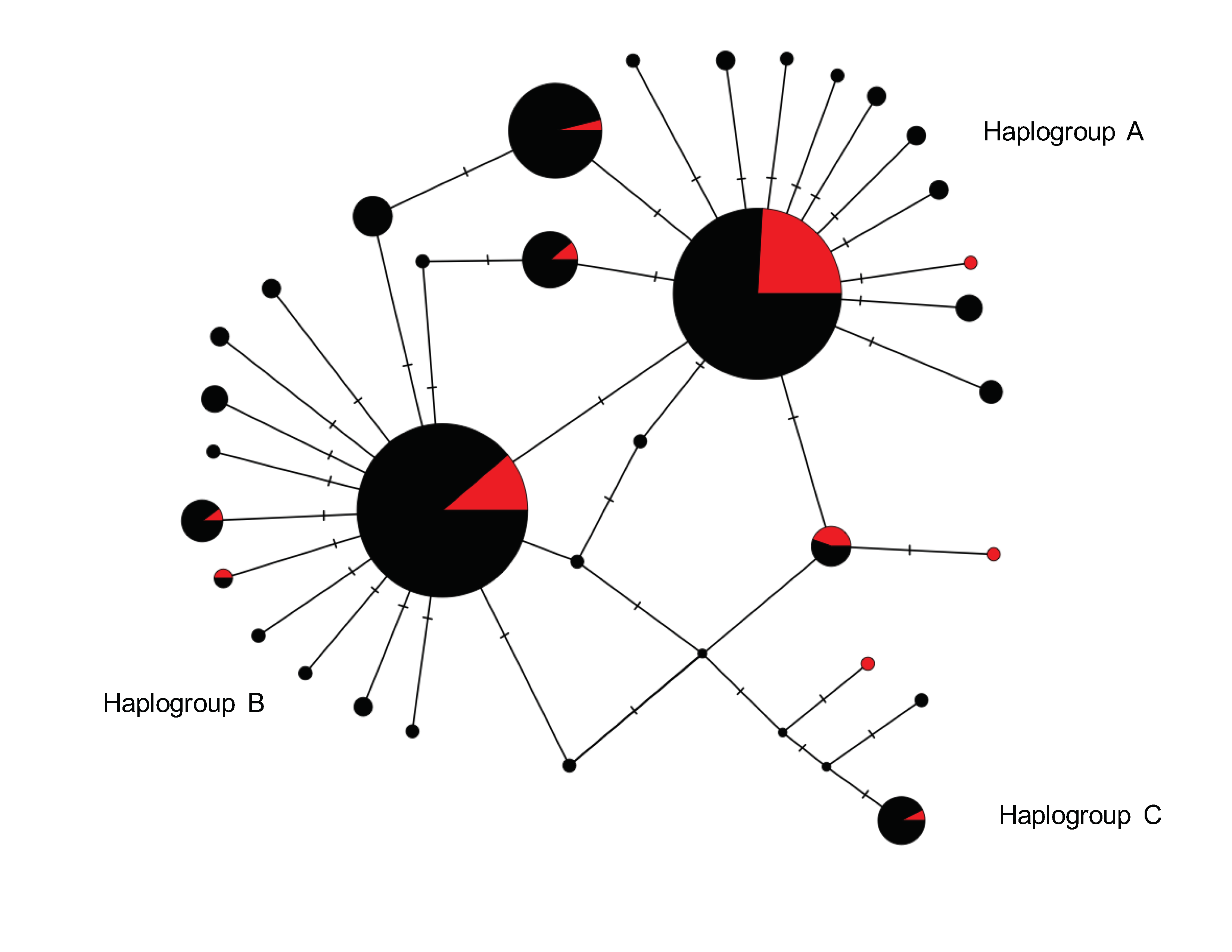

Supplement: S4 Fig — The figure shows the TCS network of the COI gene and the proportion of infected individuals within the mtDNA haplogroups A, B, and C. Haplogroup A is associated with the NWGU, haplogroup B is associated with the NEGU, and haplogroup C is associated with the WGU. Red represents individuals with Spiroplasma infection while black represents the uninfected ones. The circle size represents the number of individuals sharing a haplotype; small black nodes represent inferred haplotypes; dashes between haplotypes represent a single mutational step. The haplotype network was generated using the TCS method [55] available in POPART [56]. (TIF) [file pntd.0007340.s004.tif]
