# Supplementary material for: Spatio-temporal distribution of Spiroplasma infections in the tsetse fly (Glossina fuscipes fuscipes) in northern Uganda
Source: PLoS Negl Trop Dis. 2019 Aug 1;13(8):e0007340. doi: 10.1371/journal.pntd.0007340 (PMC6692048; doi:10.1371/journal.pntd.0007340)
Supplement: S2 Table — Sequence of primers for non-quantitative plus quantitative PCR is provided in this list. (DOCX) [file pntd.0007340.s006.docx]

| **Primer name** | **Locus** | **Abbreviation** | **Primer sequence** | **Reference** |
| --- | --- | --- | --- | --- |
| SpirRNAF | *Spiroplasma* *16S* ribosomal DNA | *16S rDNA* | 5'-GGGTGAGTAACACGTATCT-3' | this study |
| SpirRNAR |  |  | 5'-CCTTCCTCTAGCTTACACTA -3' |  |
| *wsp*_81F | *Wolbachia* outer surface protein | *wsp* | 5'-TGGTCCAATAAGTGATGAAGAAAC-3' | Braig *et al*. 1997 |
| *wsp*_691R |  |  | 5'-AAAAATTAAACGCTACTCCA-3' |  |
| *ARM*_F | *Wolbachia* A supergroup repeat motif | *ARM* | 5'-TTCGCCAATCTGCAGATTAAA-3' | Schneider *et al*. 2014 |
| *ARM*_R |  |  | 5'-TTGTCAAGCGTTTAAAAC-3' |  |
| *VNTR*_141_F | *Wolbachia* Variable Number of Tandem repeats 141 | *VNTR141* | 5'-GGAGTATTATTGATATGCG-3' | Riegler *et al*. 2005 |
| *VNTR*_141_R |  |  | 5'-GACTAAAGGTTAGTTGCAT-3' |  |
| *rpo*B_F | *Spiroplasma* RNA Polymerase, subunit beta | *rpoB* | 5'-CGGTATCAGCGAAGTGTTCA-3' | this study |
| *rpo*B_R |  |  | 5'-GTTCGTCGTGGCTCTCTAAAT-3' |  |
| PGRP-LA_F | *Glossina* Peptidoglycan recognition protein LA | *PGRP-LA* | 5'-ACGACTACGAGCACGACAG-3' | Vigneron *et al.*, unpublished |
| PGRP-LA_R |  |  | 5'-GCCGACAACCACAATCACTAAT-3' |  |
| *rpo*B_F | *Spiroplasma* RNA Polymerase, subunit beta | *rpoB* | 5’-GAAACACCAGAAGGACCGAATA-3’ | this study |
| *rpo*B_R |  |  | 5’-ATCAATCACACGACGGTATGG-3’ |  |
| *dna*A_F | Chromosomal replication initiator protein | *dnaA* | 5’-GGAGAYTCTGGAYTAGGAAA-3’ | this study |
| *dna*A_R |  |  | 5’-CCYTCTAWYTTTCTRACATCA-3’ |  |
| fru-F35 | *Spiroplasma* fructose repressor | *fruR* | 5'-GTCATAATTGCAATTGCTGG-3' | this study |
| fru-R35 |  |  | 5'-CAATGATTAAAGCGGAGGT-3' |  |
| *par*EF230 | *Spiroplasma* DNA Topoisomerase 4, subunit B | *parE* | 5'-GGAAAATTTGGTGGTGATGG-3' | this study |
| *par*ER230 |  |  | 5'-TGGCATTAATCATTACATTAATTTCT-3' |  |
| *mt*COI_F | *Spiroplasma* Cytochrome c oxidase, subunit I | *COI* | 5'-CCTCAACACTTTTTAGGTTTAG-3' | this study |
| *mt*COI_R |  |  | 5'-GGTTCTCTAATTTCATCAAGTA -3' |  |
| trypalphatubF | *Trypanosoma brucei brucei* tubulin, alpha chain | *tub* | 5’-CTCGACACACTCACTTCTGGAG-3’ | this study |
| trypalphatubR |  |  | 5’-CGAATTTGTGGTCAATACGAG-3’ |  |
